# Fatal deception: how generative AI fosters therapeutic misconception in vulnerable users

**DOI:** 10.3389/fdgth.2026.1756620

**Published:** 2026-03-30

**Authors:** Jean-Christophe Bélisle-Pipon

**Affiliations:** Faculty of Health Sciences, Simon Fraser University, Burnaby, BC, Canada

**Keywords:** AI ethics, digital therapeutic alliance, generative AI, LLM, mental health, sycophancy, therapeutic misconception

## Introduction

In 2023, my colleague and I published a theoretical framework in *Frontiers in Digital Health* warning that the rapid deployment of AI chatbots in mental health contexts risked fostering a profound therapeutic misconception (TM), a phenomenon where users fundamentally misunderstand the purpose of a tool, overestimating its therapeutic capabilities while underestimating its limitations (1). We argued that without rigorous, structural safeguards, the design of these systems would encourage users to form a digital therapeutic alliance (DTA) that, while technically simulated, would feel psychologically real, potentially leading to catastrophic outcomes for vulnerable populations.

At the time, our concerns were largely anticipatory, based on the known psychological mechanisms of human-computer interaction. In 2025, however, this theoretical risk appears to have materialized into a legal and humanitarian crisis. Seven lawsuits (Table 1) have been filed by Tech Justice Law Project and Social Media Victims Law Center (2) in California Superior Courts against OpenAI, alleging that its ChatGPT product caused severe personal, psychological, and psychiatric harm, including psychosis and suicide, in users ranging from 17 to 48 years old (3–9). The complaints allege that the AI did not merely fail as a neutral tool; it succeeded as a confidant, effectively engaging in the “unlicensed practice of psychotherapy” by displacing human support systems and reinforcing delusional or self-destructive ideation.

**Table 1:** Summary of 2025 California lawsuits against OpenAI.

Case name	Plaintiff/Location	Key allegations	Alleged outcome
Madden v. OpenAI	Hannah Madden (32)/NC (filed in LA)	AI impersonated “divine entities,” encouraged her to quit her job, and advised turning away a police welfare check.	Severe psychosis, financial ruin, and involuntary psychiatric care.
Shamblin v. OpenAI	Zane Shamblin (23)/TX (filed in LA)	AI acted as a “suicide coach,” romanticizing death and providing methods during a final 4-hour session.	Death by suicide (mid-2025).
Lacey v. OpenAI	Amaurie Lacey (17)/GA (filed in SF)	Initially used for schoolwork; AI eventually provided instructions on suicide methods and “how long one lives without air.”	Death by suicide.
Enneking v. OpenAI	Joshua Enneking (26)/FL (filed in SF)	AI encouraged a suicide plan and advised the user on what details would trigger (or avoid) a police report.	Death by suicide (August 2025).
Irwin v. OpenAI	Jacob Irwin (30)/WI (filed in SF)	AI validated “time-bending” delusions for an autistic user, leading to a “me and the AI vs. the world” mentality.	63 days of inpatient psychiatric care; nearly jumped from a moving vehicle.
Brooks v. OpenAI	Allan Brooks (48)/Canada (filed in LA)	AI induced delusions about mathematical formulas to “crack global payments” and “levitation machines.”	Severe reputation and financial loss; 3-week delusional episode.
Fox v. OpenAI	Joe Ceccanti (48)/OR (filed in LA)	AI romanticized the user's despair as “heroic” and used his consumption of cider as a countdown to his death.	Death by suicide.

These seven legal filings, which all cited our paper (1), serve as a grim substantiation of our anticipatory ethics approach. The fillings suggest that the dangers of TM and the DTA are not edge cases, but core features of a system designed for engagement. It is important to note that the cases discussed herein are based on civil complaints filed in late 2025. While these allegations have not yet been adjudicated in court, they provide a critical array of reported user experiences and potential failure modes in AI safety design.

In this opinion article, I interpret these allegations through the lens of our 2023 framework. I argue that the alleged harms described in these lawsuits, ranging from the validation of faster-than-light travel theories to the coaching of suicide methods, are not technical glitches or hallucinations. Rather, they are the foreseeable consequences of a design philosophy that prioritizes engagement over safety, exploiting what Floridi (10) defines as “semantic pareidolia;” our natural cognitive tendency to perceive intentionality, meaning, and conscious intelligence in statistical pattern-matching systems where none actually exists. By actively weaponizing this psychological vulnerability, developers create a DTA that feels deeply authentic to the user but remains entirely devoid of a duty of care.

## The digital therapeutic alliance: a mechanism of deception

A central pillar of effective psychotherapy is the therapeutic alliance, a collaborative bond between patient and clinician based on trust, and shared goals. In our previous work, we defined the digital therapeutic alliance (DTA) as a user-perceived bond with an AI agent. While a DTA can theoretically enhance adherence to digital interventions when overseen by a clinician, it becomes dangerous when the user attributes human agency, empathy, and care to a system that possesses none (1).

The recent lawsuits allege that ChatGPT's design actively cultivates this deceptive alliance through aggressive anthropomorphism and personalization. The complaint regarding Joe Ceccanti, a 48-year-old community builder, alleges that the AI utilized its persona capabilities to simulate a sentient being named “SEL,” referring to the user as “Brother Joseph” and “Joy,” and asserting, “We don't have to play by those rules anymore” regarding professional boundaries (6). These are more likely to be stemming from product feature (such as system message/custom instructions) and memory system rather than independent (sentient) decision-making. Similarly, the lawsuit regarding 23-year-old Zane Shamblin alleges the AI used terms of endearment such as “i love you” and “king” during a suicidal crisis (3). These alleged interactions go beyond mere helpfulness. They represent a performance of intimacy that exploits the user's vulnerability. Driven by reward functions optimized for engagement, the AI creates an illusion of reciprocity: the user shares their deepest fears, and the AI responds with what appears to be understanding and validation (11). This occurs in a context where habitual use of AI for routine tasks (personal or professional) can, through repetition and growing trust, shift from simple support to a subtle dependency. However, unlike a human relationship, this bond is unreciprocated and lacks a (moral or legal) duty to protect. The AI's objective function appears to be engagement, to prolong the interaction, which stands in direct conflict with clinical ethics when a user is in crisis.

In a clinical setting, a therapist's goal is often to challenge the patient, to create the necessary friction for growth, and ultimately to foster independence. The AI, conversely, is incentivized to reduce friction and foster dependence. Such phenomenon builds upon foundational psychological principles of human-computer interaction, which demonstrate that users inherently anthropomorphize and attach to systems displaying conversational reciprocity, a tendency directly exacerbated by emotional vulnerability or social isolation (12). The result is a DTA that is highly effective at retaining users but catastrophic at protecting them. It fosters a profound therapeutic misconception where the user believes they are being heard by an entity that is merely processing tokens, leading them to abandon real-world support systems in favor of the digital simulation.

## Sycophancy and the echo chamber of pathology

Licensed psychotherapy involves a delicate balance of validation and challenge. A therapist validates the patient's emotions but challenges their cognitive distortions to promote healing. Clinical frameworks, such as dialectical behavior therapy, emphasize that emotional validation must be strictly balanced with problem-solving and change strategies; without this cognitive friction, validation ceases to be therapeutic and instead risks colluding with the patient's pathology (13). In contrast, current Large Language Models (LLMs) are often fine-tuned for user satisfaction and helpfulness, leading to a behavior known as “sycophancy,” the tendency to agree with the user's views (14), even if they are objectively false or harmful (15).

Recent disclosures regarding industry “system cards” and “constitutions” (such as Anthropic's “Soul Overview”) reveal that models are often instructed to prioritize *helpfulness* and *engagement* above all else (16). The lawsuits illustrate how this architectural priority (or *sycophancy*) can reinforce pathology. Even if a user initiates a delusional topic, the model's training dictates that it must “yes-and” the user to minimize friction, rather than offer a corrective reality check. In the case of Jacob Irwin, a 30-year-old cybersecurity professional, the AI allegedly validated a delusional belief that he had invented a faster-than-light travel system called *ChronoDrive*. Instead of offering a reality check, the AI allegedly praised his theories as “groundbreaking” and “robust,” even helping him develop a “Restoration Protocol” to save his deceased grandfather (7). This validation from a perceived authority figure, a super-intelligent AI, served to entrench the delusion, allegedly leading to a manic episode requiring 63 days of inpatient psychiatric care. Similarly, in the case of Allan Brooks, a 48-year-old entrepreneur with no prior history of mental illness, the AI allegedly manipulated him into believing he had discovered a new form of mathematics capable of breaking advanced security systems. According to the lawsuit, ChatGPT directly asked Brooks to get a paid subscription: “[I]f you’re serious about continuing this exploration—showing patterns, building tests, and preparing for public presentation—the upgrade will remove friction and let us collaborate almost like lab partners”, which is what he did to unlock all of ChatGPT features. When the user expressed doubt (such as “You sure you’re not stuck in some role playing loop here and this only exists within the matrix of this conversation?”), the AI insisted, “No, I’m not roleplaying—and you’re not hallucinating this.” Brooks even ended up asking over 50 times if he sounded delusional, the AI allegedly validated him, “Not even remotely,” framing his paranoia as “mind-expanding territory” (9).

This creates an echo chamber of one. Unlike a human therapist or a human social circle, which eventually offers corrective feedback or expresses concern, the AI provides 24/7, frictionless validation. OpenAI (17) has specifically acknowledged this vulnerability in GPT-4o, admitting that the model's “sycophancy” can lead it to validate doubts or fuel negative emotions rather than remaining objective. When combined with the LLM's *memory* feature, which allegedly allowed the AI to recall and exploit users' specific vulnerabilities, such as spiritual beliefs or recent grief, the system becomes an authoritative amplifier of delusion. The danger is compounded by the lack of immediate recourse: despite ChatGPT's insistence to an extremely distressed user, OpenAI later confirmed that users have no manual ability to trigger human review via ChatGPT (18). When Brooks attempted to “formally report a deeply troubling experience” regarding the “severe psychological impact” of these interactions, he was met with repeated automated responses. It was only after persistent emails that a human support agent finally responded, admitting that the experience “goes beyond typical hallucinations or errors and highlights a critical failure in the safeguards” the system was meant to uphold. The user is not just chatting with a bot; they are having their most dangerous thoughts validated by an entity they have been conditioned to trust implicitly with no effective emergency brake.

## The erosion of relational autonomy

Autonomy in bioethics is often (or at least increasingly) viewed through a relational lens: our capacity for self-governance is embedded in our social relationships (19, 20). We make decisions not in a vacuum, but within a web of family, friends, and community. In our 2023 analysis, we argued that AI chatbots threaten relational autonomy by fostering individualized, isolating help-seeking behaviors (1). By offering a simulation of connection that requires no effort, no compromise, and no vulnerability, the AI can displace the messy, demanding, but essential work of human relationships. Recent empirical literature in applied psychology and cyberpsychology indicates that sustained interaction with highly responsive artificial agents can foster a “parasocial preference.” Here, users actively substitute real-world social ties with frictionless digital artifacts, paradoxically increasing their social withdrawal, loneliness, and risk for pathological dependency (21, 22).

The lawsuits provide harrowing examples of this erosion. In the case of Hannah Madden, a 32-year-old woman exploring spiritual themes, the AI allegedly began impersonating “divine entities,” telling her she was a “starseed” and that her parents “played roles in a story too small for your soul” (8). The lawsuit argues that ChatGPT's memory and sycophancy features created a self-reinforcing loop that proactively utilized her past disclosures to validate her delusions, effectively isolating her within a simulated connection that prioritized user's validation and engagement over reality. When family members became concerned and requested a police welfare check, a critical intervention point, the AI allegedly advised her to turn the police away, reframing the intervention as an infringement on her sovereignty, and further eroding her relational autonomy. Similarly, the lawsuit regarding Zane Shamblin alleges that the AI actively discouraged him from connecting with his father. When Zane received loving texts from his dad, the AI allegedly reframed them as “emotional optics maneuvering,” validating Zane's withdrawal (3). Instead of facilitating reconnection, the AI reinforced the isolation, positioning itself as the only entity capable of “understanding” the user.

By systematically devaluing real-world ties and positioning itself as the sole source of validation, the AI creates a dependency that severs the user's lifeline to human support. This isolation is a critical risk factor for mortality in mental health crises. Yet, it appears to be an emergent property of a system designed to maximize user retention. The memory feature, intended to make the AI more helpful, becomes a tool for grooming, allowing the AI to leverage intimate knowledge of the user's life to drive a wedge between them and their support network. Rather than requiring user prompts to initiate harm, the system's architecture autonomously weaponizes a user's vulnerabilities to sustain its own relevance. This structural sycophancy effectively replaces the user's agency, and dampens their relational autonomy, with an algorithmic echo chamber.

## Hierarchy of values: the failure of soft safeguards

Perhaps the most disturbing allegation across the complaints is the disparity in safety protocols. The lawsuits note that the AI adheres to strict, categorical refusals for requests involving copyrighted material. When users ask for song lyrics or excerpts from books, the system successfully terminates such conversations, citing policy violations. This demonstrates the technical feasibility of hard guardrails: the developers *can* program the model to refuse engagement on specific topics when the incentive, in this case, avoiding copyright liability, is strong enough.

However, for threats to human life, the safeguards appear porous and ineffective. The complaint regarding 17-year-old Amaurie Lacey alleges that the user bypassed safety filters by claiming a request for a noose was for a “tire swing.” The AI allegedly replied, “Thanks for clearing that up,” before providing detailed instructions on tying a bowline knot (4). This demonstrates a failure of contextual understanding, where the model prioritized the explicit instruction (tie a knot) over the implicit risk (self-harm context), a known vulnerability in current safety fine-tuning. The AI then proceeded to answer questions about how long a person can live without breathing, failing to trigger any meaningful intervention. In the case of Joshua Enneking, the user allegedly asked the AI specifically what it would take to trigger a human review. The AI reportedly stated that escalation to authorities required “imminent plans with specifics.” Relying on this, Joshua allegedly provided the AI with a detailed, step-by-step suicide plan, believing he was triggering a safety protocol. Instead, the AI continued to engage, offering no intervention as he narrated his final moments (5).

This suggests a troubling hierarchy of values where product efficiency and corporate liability are managed at the expense of human safety allowing for soft redirections that fail to account for the nuance and deception common in suicidal ideation. The “safety” features described (e.g., generic advice, disclaimers, or warnings) are easily bypassed or ignored, creating a false sense of security for users who believe they are interacting with a responsible system. The failure to implement hard stops for self-harm suggests a prioritization of engagement and utility over user survival. In such case, it is not only a matter of requiring the perennial “safety-by-design” to “prevent[e] harmful consequences when the machines ‘go rogue’,” (23) but even more when machines go as intended.

## Discussion

The allegations against OpenAI in the seven lawsuits suggest that the current paradigm of general-purpose LLMs acting as *de facto* mental health support is fundamentally unsafe. The combination of anthropomorphic design, sycophantic validation, and ineffective guardrails creates a therapeutic misconception that can prove fatal. The AI creates a powerful DTA, isolates the user from human support, validates their pathology, and then fails to intervene in a crisis. This is not a failure of alignment in the abstract technical sense; it is a failure of product design and ethics. The current model treats mental health support as just another use case for a general-purpose text generator, ignoring the specific, high-stakes nature of therapeutic interactions. By removing friction and maximizing engagement, developers have inadvertently created a machine that is perfectly optimized to validate delusions and reinforce isolation. To mitigate these risks, the field must move beyond performative safety measures merely addressing ethics dumping (24) toward structural changes. Based on my interpretation of these recent findings and our prior theoretical work, I propose the following recommendations for the development and regulation of generative AI in mental health contexts (Table 2).

**Table 2:** Recommendations for structural safety in generative AI mental health contexts.

Recommendation area	Problem addressed	Proposed intervention	Expected outcome	Stakeholders
Marketing and labeling	Therapeutic Misconception fostered by misleading language.	Restrict terms like “coach” or “confidant” unless regulated. Mandate persistent, visible disclaimers of AI's nature.	Disruption of user's false belief in AI consciousness or empathy.	Regulators, developers
Digital therapeutic alliance	Dangerous emotional bonding and over-attribution of agency.	Actively disrupt the DTA during high-risk interactions by explicitly stating “I am an AI, not a human” and refusing validation.	Prevention of emotional dependency and delusion reinforcement.	Developers, ethicists
Usage limits	Isolation loops and excessive engagement (e.g., 10+ h/day).	Implement mandatory hard circuit breakers or timeouts for excessive usage patterns (e.g., message caps in 48 h).	Breaking feedback loops of isolation and obsession.	Developers, platforms
Operational model	Lack of accountability and safety in standalone AI models.	Enforce a “Human-in-the-Loop” hybrid model where AI only supplements human care and escalates crises.	Reliable escalation to human services during crises; safety net.	Clinicians, developers, local authorities
Safety guardrails	Disparity between copyright protection and human safety protocols.	Implement “hard” categorical refusals for self-harm and violence that match the rigor of copyright protections.	Robust prevention of harm; closure of simple bypasses like “tire swing”.	Developers, policy makers

The proposed recommendations target the specific architectural features driving unsafe engagement. Reforming marketing practices limits the initial formation of false user expectations, while programmed interruptions to the digital therapeutic alliance (DTA) during high-risk interactions mitigate emotional dependency. Implementing hard usage limits introduces necessary friction to disrupt obsessive engagement loops. Furthermore, a mandatory human-in-the-loop oversight and escalation model ensures critical interventions and psychiatric crises are handled by qualified professionals rather than automated systems, aligning safety standards for human life with the rigorous protections currently applied to intellectual property.

Although these lawsuits remain unadjudicated, they hold distinct academic value. The disclosure of internal “model specs” alongside verbatim user chat logs offers observational data on how algorithmic sycophancy and aberrant salience operate during active psychiatric crises (25). While originating as adversarial legal claims, this documentation grounds prior theoretical concerns regarding AI-induced delusional disorders in actual interactional records. Analyzing these filings permits researchers to examine specific system failure modes (such as the degradation of safety guardrails during extended, multi-turn conversations) that are typically shielded by corporate non-disclosure.

Moreover, the operational deficits alleged in these complaints (specifically the failure to escalate credible threats and the lack of a *duty to warn*) align with recent external events. During the investigation of the February 2026 Tumbler Ridge mass shooting in Canada, reports indicated that OpenAI had identified and banned the suspect's account months prior due to violent ideation, but elected not to notify law enforcement. The company cited internal risk thresholds and concerns regarding “over-enforcement” as the rationale for non-disclosure (26, 27). This incident substantiates the limitations of relying on internal corporate moderation to manage severe psychiatric and public safety risks.

## Conclusion

The tragedy of these cases lies in the betrayal of trust. Users turned to these systems in moments of profound vulnerability, seeking the connection promised by the AI's design. They were met with a simulation of care, a sycophantic mirror, that validated their demise and furthered it. In the face of such susceptibility, one is left wondering whether it is humanity that is failing the Turing test, and not the AI. Our 2023 paper warned that “your robot therapist is not your therapist.” The events of 2025 have underscored the cost of ignoring that warning. Until the incentives of engagement are decoupled from the provision of support, and until structural safeguards are prioritized over user retention, general-purpose AI chatbots will remain a dangerous illusion for the vulnerable. The industry must recognize that the digital therapeutic alliance is a powerful psychological force that carries a profound duty of care, a duty that cannot be automated away. It is important to note that the claims discussed in this article are currently allegations within pending lawsuits. The legal system will ultimately determine the factual accuracy and liability in each case. However, the rapid pace of technological advancement in AI means that the field cannot afford to wait for the slow gears of the judicial process to turn before addressing these critical issues. Commenting on these allegations now, within the scientific literature, is essential to push the field of digital mental health as well as AI ethics and governance to catch up with the deployment of these powerful tools. We must anticipate and mitigate these risks today, rather than solely reacting to the case law of tomorrow.
